# Autoantibodies as biomarkers for breast cancer diagnosis and prognosis

**DOI:** 10.3389/fimmu.2022.1035402

**Published:** 2022-11-14

**Authors:** Ruozhu Yang, Yi Han, Wenjun Yi, Qian Long

**Affiliations:** Department of General Surgery, The Second Xiangya Hospital of Central South University, Changsha, China

**Keywords:** breast cancer, autoantibody, early diagnosis, prognosis, autoantibody detection

## Abstract

Breast cancer is the most common cancer in women worldwide and is a substantial public health problem. Screening for breast cancer mainly relies on mammography, which leads to false positives and missed diagnoses and is especially non-sensitive for patients with small tumors and dense breasts. The prognosis of breast cancer is mainly classified by tumor, node, and metastasis (TNM) staging, but this method does not consider the molecular characteristics of the tumor. As the product of the immune response to tumor-associated antigens, autoantibodies can be detected in peripheral blood and can be used as noninvasive, presymptomatic, and low-cost biomarkers. Therefore, autoantibodies can provide a possible supplementary method for breast cancer screening and prognosis classification. This article introduces the methods used to detect peripheral blood autoantibodies and the research progress in the screening and prognosis of breast cancer made in recent years to provide a potential direction for the examination and treatment of breast cancer.

## Introduction

Breast cancer is the most common cancer in women. There were approximately 2.3 million new cases and 685,000 deaths due to breast cancer worldwide in 2020 (1). In the United States, 290,560 new cases and 43,780 deaths have been estimated to occur in 2022 (2). Early diagnosis is vital to improve the survival rate of breast cancer. The five-year relative survival rate for breast cancer in the United States increased from 79% between 1984 and 1986 to 91% between 2008 and 2014, largely due to improvements in early diagnosis and treatment (3).

The most common method used to screen for breast cancer is mammography. Current guidelines all recommend annual or biennial mammography starting at age 40 (4–6). Several studies have shown significant decreases in mortality from breast cancer among women who undergo mammography, with an average reduction of 40 to 46% (7–9). Mammography, however, has many drawbacks. Data from Vermont in the US and Norway yielded sensitivities of 88.2% and 90.7%, respectively (10). According to a study from the Netherlands, the sensitivity of mammography was 85% in individuals with 20 mm-sized breast tumors and was even lower in individuals with smaller breast tumors (11). Inadequate sensitivity can lead to a considerable number of missed diagnoses. Mammography can also lead to false positives or overdiagnosis, causing unnecessary treatment and psychological distress (12, 13). In the United States, 23.8% of women who receive regular mammography had at least one false positive over a 10-year period (14). A Canadian study found that annual mammography did not reduce the mortality rate due to breast cancer in women ages 40 to 59, and 0.24% of those who participated were overdiagnosed with invasive breast cancer (15). In addition, a high density of breast tissue is an independent risk factor for breast cancer, and mammography is less sensitive in high-density breast tissue (16–18). Younger women tend to have denser breasts, which also makes mammography less sensitive (19). These patients can undergo additional ultrasound or magnetic resonance imaging (MRI) to improve sensitivity, but the false positive rate also increases (20).

Tumor, node, and metastasis (TNM) staging is the most used method to determine the prognosis of breast cancer. This system includes an assessment of the characteristics of the primary tumor, regional lymph nodes, and distant metastasis (21). However, this method does not reflect the molecular characteristics of the tumor and does not enable a more accurate prognostic analysis based on the molecular heterogeneity of cancer cells (21). Breast cancer pathological types, molecular subtypes, and gene expression features, including risk alleles, methylation, and single nucleotide polymorphisms, also contribute to different outcomes (22–25). According to assessments of different molecular characteristics and prognoses, including assessments of recurrence risk and survival rate, individualized treatment methods that are more accurate can be adopted (26).

In cancer patients, tumor-associated antigens (TAAs) produced by tumor cells activate B cells that can produce autoantibodies to TAAs. Through the amplification effect of humoral immunity, autoantibodies in the peripheral blood of patients are far more abundant than TAAs, and autoantibodies also have longer half-life. Therefore, by detecting autoantibodies in peripheral blood, breast cancer patients can be screened in the early stage and their prognosis can be predicted. At present, this method is not as effective as mammography, but as a complementary examination, it can help improve the sensitivity and specificity of breast cancer screening and establish more accurate prognostic analysis method. Therefore, the detection of autoantibodies has a promising prospect in the future. This review includes a discussion of the advances in autoantibodies detected in peripheral blood in the diagnosis and prognosis of breast cancer.

## Autoantibodies in breast cancer

**Figure 1:**
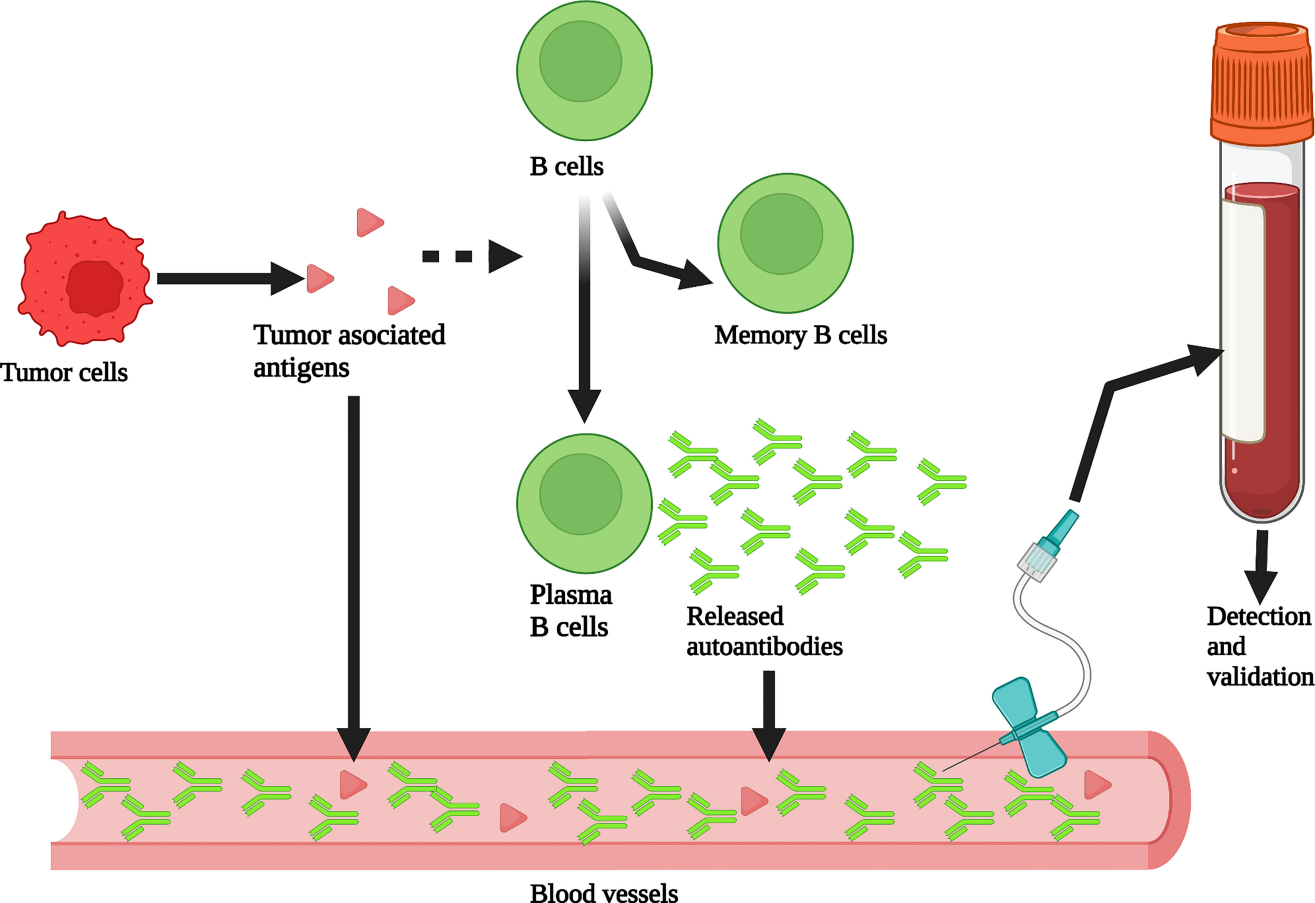
Production and detection of peripheral blood autoantibodies. Tumor associated antigens (TAAs) produced by tumor cells stimulate B cells to differentiate into memory B cells and plasma B cells that can produce autoantibodies. These autoantibodies enter the peripheral blood. The abundance of autoantibodies in peripheral blood is much greater than that of TAAs through the amplification of the immune response process. Therefore, autoantibodies can be detected from plasma or serum to assist in breast cancer screening. Created with BioRender.com.

The tumor microenvironment plays a decisive role in the occurrence, development, and treatment of tumors (27). Due to somatic mutations and genomic instability, the proteome of tumor cells is modified by phosphorylation, acetylation, and glycosylation, resulting in tumor-associated antigens (TAAs). The body produces autoantibodies when TAAs are recognized by the immune system (28). The BCR on B cells specifically binds to the TAAs to initiate an antigen stimulation signal, which is co-transmitted by BCR- Igα/Igβ and CD19/CD21/CD81. As antigen-presenting cells, B cells internalize and process BCR binding antigens through endocytosis. The antigenic peptide produced by antigen degradation binds to MHC class II molecules and is presented to specific Th cells. Activated Th cells express CD40L, which provides a second signal for B cell activation. Activated B cells then produce specific autoantibodies which are released into the peripheral blood. These autoantibodies can be used as new tumor detection indicators to predict the occurrence and prognosis of tumors (**Figure 1**). Detecting autoantibodies has many advantages. First, autoantibodies have higher stability than other serum proteins and are not easy to hydrolyze, with a half-life of 7-30 days. Second, autoantibodies can be detected when the tumor has developed but clinical symptoms have not yet appeared (28–30). Third, the detection of autoantibodies in peripheral blood is not affected by the density of breast tissue (31, 32); therefore, mammography defects can be prevented using this approach. Finally, peripheral blood autoantibodies can better reflect the molecular characteristics and heterogeneity of breast cancer. The analysis of most autoantibodies individually lacks sufficient sensitivity (33–35), so much research has focused on the analysis of autoantibody combinations. At present, autoantibodies can be used to predict the early occurrence and prognosis of breast cancer; however, this analysis is still in the early stage of development, and there have been no clinical trial reports. Most of the studies are in phase 1 (preclinical exploratory phase) or phase 2 (clinical testing and validation phase of biomarker development) (36–39).

## Autoantibodies as biomarkers for breast cancer

**Tables 1**, **2** summarize the discovery and application of peripheral blood autoantibodies and autoantibody panels in the diagnosis and prognosis of breast cancer in recent years. Autoantibodies such as p53, MUC1, and HER2/NEU were discovered many years ago and have been studied in detail.

**Table 1:** Autoantibodies in the Diagnosis of Breast Cancer.

Antibodies/Antigens	pe of study	Source	Cohort			Methodology		Diagnostic value					Reference	Year
			Sample type	Discovery set	Validation/test set	Discovery set	Validation/test set	Groups compared	Biomarkers analyzed	AUC	Sensitivity (%)	Specificity (%)		
FTH1 + hnRNPF + CA 15-3	R	serum	BC	155	NA	ELISA	NA	BC *vs*. HC	FTH1 + hnRNPF + CA 15-3	0.931	89.3	93.8	(40)	2013
			HC	155	NA									
			ONBC	49	NA									
K94p1	R	serum	BC	NA	30	NA	Phage ELISA	BC *vs*. HC	K94p1	0.648	50	82.6	(41)	2013
			HC	NA	30									
GAL3+RACK1+PAK2+PHB2+RUVBL1	R	serum	DCIS	10	55	proteomic analysis	ELISA	HC *vs*. DCIS+T1N0PBC	GAL3+RACK1+PAK2+PHB2+RUVBL1	0.81	66	87	(42)	2013
			T1N0PBC	10	59									
			BBD	20	NA			HC *vs*. T1N0PBC	GAL3+RACK1+PAK2+PHB2+RUVBL1	0.81	66	84		
			HC	20	68									
			RA	10	NA			HC *vs*. DCIS	GAL3+RACK1+PAK2+PHB2+RUVBL1	0.85	82	74		
			SLE	10	NA									
ALDH7A1+ALDOA+ DLD + ENO1 + FBP1 + GAPDH + GPI + PKM2+ TPI1 + EFTUD2 + HNRNPA1 + HNRNPA2B1 + HNRNPK + HSPA8+ PTBP1 + RALY + SAP18 + SF3A1 + SFRS1+ SFRS3 + SFRS6 + SYNCRIP+ U2AF1	P	plasma	ER+/PR+BC	48	NA	Protein Microarray	NA	HC *vs*. ER+/PR+BC	ALDH7A1+ALDOA+ DLD + ENO1 + FBP1 + GAPDH + GPI + PKM2+ TPI1 + EFTUD2 + HNRNPA1 + HNRNPA2B1 + HNRNPK + HSPA8+ PTBP1 + RALY + SAP18 + SF3A1 + SFRS1+ SFRS3 + SFRS6 + SYNCRIP+ U2AF1	0.77	35	95	(43)	2013
			HC	65	NA									
Sixteen models, each including age and four autoantibodies; or Antigens no.016+080+095+115	R	serum	BC	NA	201	NA	ELISA	ONBC&HC&LCIS *vs*. BC	Sixteen models, each including age and four autoantibodies	0.801	95.2	49.5	(44)	2013
			ONBC, HC, LCIS	NA	345			ONBC&HC&LCIS *vs*. BC	Antigens no.016+080+095+115	0.845	94.7	61.8		
c-myc+survivin+cyclin B1+cyclin D1 +p62+p53+p16+CDK2	R	serum	BC	NA	41	NA	ELISA	BC *vs*. HC	c-myc+survivin+cyclin B1+cyclin D1 +p62+p53+p16+CDK2	NA	61	89	(45)	2013
			HC	NA	82									
ANGPTL4 + DKK1 + GAL1 + GFRA1 + GRN + LRRC15 + MUC1 (+ age, BMI, race and current smoking status)	R	plasma	BC	200	NA	ELISA	NA	BC *vs*. HC	ANGPTL4 + DKK1 + GAL1 + GFRA1 + GRN + LRRC15 + MUC1 (+ age, BMI, race and current smoking status)	0.818	72.9	76	(46)	2014
			HC	200	NA									
HSP60+FKBP52+PRDX2+PPIA+MUC1+GAL3+PAK2+PHB2+RACK1+RUVBL1+p53+HER2+CCNB1	R	serum	DCIS	NA	87	NA	ELISA	HC *vs*. DCIS+T1N0PBC	HSP60+FKBP52+PRDX2+PPIA+MUC1+GAL3+PAK2+PHB2+RACK1+RUVBL1+p53+HER2+CCNB1	NA	90	42	(47)	2014
			T1N0PBC	NA	153			HC *vs*. T1N0PBC		NA	90	51		
			HC	NA	156			HC *vs*. DCIS		NA	90	32		
Imp1+p16+Koc+survivin+cyclin B1+ c-myc	R	serum	BC	NA	49	NA	ELISA, WB	BC *vs*. HC	Imp1+p16+Koc+survivin+cyclin B1+ c-myc	NA	67.3	92.2	(48)	2015
			BBT	NA	35									
			HC	NA	38									
IMP2/p62	R	serum	BC	NA	49	NA	ELISA, WB, Indirect immunofluorescence	BC *vs*. BBT	IMP2/p62	0.714	NA	NA	(49)	2015
			BBT	NA	36			BC *vs*. HC	IMP2/p62	0.615	NA	NA		
			HC	NA	44									
TP53	R	serum	HRNBC	NA	43	NA	ELISA	HRNBC *vs*. HC	TP53	0.677	34.9	90	(50)	2015
			HC	NA	87			TN *vs*. HC	TP53	0.632	35.7	90		
CTAG1B+CTAG2+TP53+RNF216+PPHLN1+PIP4K2C+ZBTB16+TAS2R8+WBP2NL+DOK2+PSRC1+MN1+TRIM21	R	plasma	BLBC	45	145	Protein array	ELISA	BLBC *vs*. HC	CTAG1B+CTAG2+TP53+RNF216+PPHLN1+PIP4K2C+ZBTB16+TAS2R8+WBP2NL+DOK2+PSRC1+MN1+TRIM21	0.68	33	98	(36)	2015
			HC	45	145									
TYMS, PDLIM1	R	plasma	BC	30	64	SERPA	ELISA	BC *vs*. HC+RA	TYMS	0.804	57.8	95	(51)	2016
			HC	30	50				PDLIM1	0.711	73.4	58.3		
			RA	NA	10									
FRS3+RAC3+HOXD1+GPR157+ZMYM6+EIF3E+CSNK1E+ZNF510+BMX+SF3A1+SOX2	R	serum	HC	NA	18	NA	ELISA	BC *vs*. HC+BBD	FRS3+RAC3+HOXD1+GPR157+ZMYM6+EIF3E+CSNK1E+ZNF510+BMX+SF3A1+SOX2	0.77	72.2	70.8	(52)	2016
			BBD	NA	92									
			BC	NA	100									
HSPB1+HSPD1+HSP70+p53+HSP90+HSPA5+HSP90B1	P	serum	BC	NA	50	NA	Protein Microarray	BC *vs*. HC	HSPB1+HSPD1+HSP70+p53+HSP90+HSPA5+HSP90B1	0.978	86	100	(53)	2016
			HC	NA	26									
LGALS3+PHB2+MUC1+GK2+CA15-3	R	serum	BC	10	100	SEREX	Protein Microarray, WB	BC *vs*. HC	LGALS3+PHB2+MUC1+GK2+CA15-3	0.872	87	76	(54)	2016
			HC	5	50									
RAD50+PARD3+SPP1+SAP30BP+NY-BR-62+NY-CO-58	R	serum	BC	112	NA	SEREX, ELISA	NA	BC *vs*. HC	RAD50+PARD3+SPP1+SAP30BP+NY-BR-62+NY-CO-58	0.808	70	91	(55)	2017
			HC	35	NA									
p16+c-myc+ TP53+ ANXA-1	R	serum	BCS I & II	NA	57	NA	ELISA	BC *vs*. HC	p16+c-myc+ TP53+ ANXA-1	NA	33.3	90	(56)	2017
			BCS III & IV	NA	45			BCS I & II *vs*. HC	p16+c-myc+ TP53+ ANXA-1	NA	31.6	90		
			HC	NA	146			BCS III & IV *vs*. HC	p16+c-myc+ TP53+ ANXA-1	NA	33.3	90		
Thioredoxin-Like 2 Autoantibody	R	serum	BC	10	NA	human protein microarray system	dot blot	BC*vs*HC	Thioredoxin-Like 2 Autoantibody	NA	NA	NA	(57)	2018
			HC	10	NA									
p53+cyclinB1+ p16+ p62+ 14-3-3ξ	R	serum	BC	10	184	WB	ELISA	BC*vs*HC	p53+cyclinB1+ p16+ p62+ 14-3-3ξ	0.916	78.2	89	(58)	2019
			HC	10	184			BC*vs*BBD		0.849	69.5	84.4		
TOPO48	R	serum	BC	NA	68	NA	ELISA	early stage BC*vs*BBD+HC	TOPO48	0.801	100	76	(59)	2020
			HC		136									
			BBD		10									
p53+RalA+p90+NY-ESO-1+HSP70+c-myc+galectin-1+Sui1+KN-HN-1+HSP40+PrxVI+p62+cyclin B1+HCC-22-5+annexinII+HCA25a+HER2	R	serum	BC	386	NA	ELISA	NA	BC*vs*HC	p53+RalA+p90+NY-ESO-1+HSP70+c-myc+galectin-1+Sui1+KN-HN-1+HSP40+PrxVI+p62+cyclin B1+HCC-22-5+annexinII+HCA25a+HER2	NA	NA	NA	(60)	2021
			HC	73										
BRCA2+CEBPA+CEP55+FUBP1+HRAS+RalA	R	serum	BC	NA	279	NA	ELISA	BC*vs*HC+ BBD	BRCA2+CEBPA+CEP55+FUBP1+HRAS+RalA	0.916	78.9	90.2	(61)	2021
			HC	NA	279	NA		BC*vs*BD		0.884	71.2	90.5		
			BBD	NA	200	NA								
BMI-1+HSP70+NY-ESO-1+p53	R	serum	BC	NA	123	NA	ELISA	BC*vs*HC	BMI-1+HSP70+NY-ESO-1+p53	0.819	63.4	90.2	(62)	2021
			HC		123									
anti- KJ901215, - FAM49B, - HYI, - GARS+- CRLF3	R	serum	ES-BC	80	245	high- density HuProtTM array, low- density focused array	ELISA	ES-BC*vs*BBD +HC	anti- KJ901215, - FAM49B, - HYI, - GARS+- CRLF3	NA	38.78	85	(63)	2022
			BBD	20	48									
			HC	19	80									

NA, Not available.

**Table 2:** Autoantibodies in the Prognosis of Breast Cancer.

Antibodies/Antigens	Type of study	Source	Cohort			Methodology		Prognostic value			Reference	Year
			Sample type	Discovery set	Validation/test set	Discovery set	Validation/testset	Groups compared	Biomarkers analyzed	Prognostic endpoint		
TPO, TG	P	serum	BC	200	NA	NA	NA	NA	TPO, TG	lower rate of axillary involvement(22% *vs*. 46%, p=0.007) and a lower rate of Ki-67 proliferation index (12.73% *vs*. 20.72%, p=0.025)	(64)	2015
HER2	P	serum	HC	100	NA	ELISA	NA	NA	HER2	higher recurrence-free survival(P = 0.015)	(65)	2016
			DCIS	100								
			IBC	500								
TOPO48	R	serum	BC	68	NA	ELISA	NA	BC	TOPO48	NA	(59)	2020
			HC	136								
			BBD	10								
SELENOP	P	serum	BC	1988	NA	ELISA	NA	NA	SELENOP	higher recurrence(HR(95%CI) = 1.87) and higher recurrence and mortality(fully adjusted HR(95%CI) per log increase of 1.25 and 1.31)	(66)	2022

AUC, area under the curve; R, retrospective study; P, prospective study; NA, not available;

ELISA, enzyme-linked immunosorbent assay; SEREX, serological analysis of recombinant cDNA expression libraries; SERPA, serological proteome analysis; WB, Western blot;

BC, patients with breast cancer; HC, healthy controls; BBD, patients with benign breast disease; DCIS, ductal carcinoma in situ; ONBC, patients with other nonbreast cancers; RA, patients with rheumatoid arthritis; HRNBC, hormone receptor-negative; ER+/PR+BC, breast cancer patients with estrogen receptor positive/progesterone receptor positive; BCS I & II, patients with breast cancer at stage I or II; BCS III & IV, patients with breast cancer at stage III or IV; BBT, patient with benign breast tumor; BLBC, patients with basal-like breast cancer; T1N0PBC, patients with primary breast cancer without lymph node metastasis at early stage; IBC, invasive breast cancer; LCIS, lobular carcinoma in situ; SLE, patients with systemic lupus erythematosus.

### p53 autoantibody

In 1979, Albert B. DeLeo et al. used Meth A antiserum to react with ^35^S methionine-labeled Meth A sarcoma and normal BALB/c lung fibroblasts. They found that Meth A sarcoma contained a protein with an apparent molecular weight (Mr) of approximately 53,000, while normal fibroblasts did not (67). This finding indicates that the Meth A antiserum contains specific antibodies against this protein. The protein is named p53. p53 can be detected in a variety of tumor cells, including breast cancer cells, in animals and humans, but not in normal cells (67–69). Therefore, p53 was initially thought to be an oncogene. However, S.J. Baker et al. subsequently found p53 mutations in the 17p region of colorectal cancer chromosomes that are not present in normal tissues (70). L.A. Donehower et al. found that p53 knockout mice developed normally but were prone to spontaneous tumors (71). Since then, p53 has been mainly studied as a tumor suppressor gene. p53 plays a role in cell cycle arrest, apoptosis, DNA repair, senescence, angiogenesis, cell metabolism, reactive oxygen species (ROS) generation, autophagy, and iron-mediated death. Its mutations can lead to the occurrence of a variety of cancers (72). In 1982, L.V. Crawford et al. detected p53 antibodies in the serum of breast cancer patients but not in healthy controls (73). In subsequent studies of p53 autoantibody detection in the peripheral blood of breast cancer patients that ended in 2016, the median sensitivity and specificity were 17.5% (4.8-100%) and 98.7% (95-100%), respectively. Meta-analysis showed that when the cutoff value was defined as the mean +2 or 3 standard deviations, the summary area under the curve (SAUC) was 0.78 (74). The analysis of p53 autoantibody levels is helpful for breast cancer screening, but because of the relatively low sensitivity, its efficacy is not ideal. However, in recent years there have been several studies using panels including p53 autoantibody, which have yielded high specificities as well as high sensitivities (53, 75). P53 and its autoantibody are widely detected to exist in tissues or serum of different kinds of cancer, and there are no studies on the specificity of p53 autoantibody in breast cancer patients so far. But combining p53 autoantibody with other autoantibodies or proteins is still helpful since p53 plays an important role in the tumorigenesis of breast cancer. Therefore, further research about p53 autoantibody is needed to develop more effective ways to screen for breast cancer. In addition, some studies have shown that the expression of serum p53 autoantibodies is associated with p53 accumulation in tissue (76). However, others speculated that there was no significant correlation between serum p53 autoantibody levels and p53 accumulation in cancer tissues, while the correlation between serum p53 protein levels and the accumulation of p53 in cancer tissues is better (77, 78). In terms of prognosis, recent studies all speculate that p53 autoantibody concentration is considerably associated with poor prognosis (79–81). Some studies have shown that p53 autoantibody concentration is significantly correlated with histological grading and other prognostic information (76, 81). However, some studies have found that p53 autoantibody concentration is not associated with the breast cancer stage (79). Moreover, the analysis of p53 autoantibodies is likely to provide little information about treatment response and tumor recurrence (82).

### MUC1 autoantibody

MUC1 is a single channel type I transmembrane protein with a highly glycosylated extracellular domain, which extends 200-500 nm from the cell surface (83–85) and is located on the surface of epithelial cells in the breast, gastrointestinal tract, respiratory tract, urinary tract and reproductive tract (86). In healthy tissues, MUC1 protects epithelial cells and acts as a barrier against pathogen colonization (87, 88). Loss of miR-125b expression in breast cancer cells leads to overexpression of MUC1 (89). MUC1 is overexpressed in 91% of breast cancers and is often found in pancreatic cancer, colon cancer, and lung cancer (86, 90). Unlike the normally expressed MUC1, ta-muc1 mainly exhibits a core 1° glycan (91) and is highly sialylated (92). Abnormal glycosylation changes induce an immune response and lead to the production of MUC1 antibodies. Using a glycopeptide microarray containing a 60-mer MUC1 glycopeptide, Blixt et al. reported that the level of MUC1 autoantibodies in patients with early breast cancer (n=365) was significantly higher than that in women with benign breast disease (n=108) or healthy controls (n=99). Interestingly, the induction of autoantibodies to the core 3 and STN glycoforms of MUC1 is significantly associated with a reduction in the incidence of metastasis and an increase in the amount of time before metastasis occurs, which may suggest that different glycoforms of MUC1 may be involved in the progression of cancer (37). A study on a BRCA1/2 mutant female population (n=127) reported that the level of MUC1 autoantibody in mutation carriers was lower than that in the healthy control group. Moreover, contrary to previous studies on women with sporadic breast cancer, no increase in MUC1 IgG antibody levels was found in women at high genetic risk of breast cancer (93). However, Burford et al. applied the 60-mer MUC1 glycopeptide and a microarray platform of recombinant MUC1 containing 16 tandem repeats to screen serum samples and verify the expression of MUC1 autoantibodies and concluded that autoantibodies against the MUC1 glycopeptide cannot be used to distinguish cases and controls (94).

### HER2/neu autoantibody

In 1984, A. L. Schechter et al. found that neuro/glioblastoma transformed with DNA from four rat neuro/glioblastoma cell lines all contained the same transforming gene, the NEU gene (95). The gene can be used to synthesize a kind of tumor antigen with a Mr of 185,000 (p185). The sequence of Neu is homologous to that of erb-B, which is the epidermal growth factor receptor (EGFR) gene, but the two are different genes (95, 96). In humans, the counterpart to neu was named HER2 (97). A subsequent study concluded that P185, a product of HER2, was found in 46% of primary breast cancers (98). In 1997, M. L. Disis et al. detected HER-2/neu antibody expression in 11% of breast cancer patients but not in normal controls using ELISA, and the presence of the HER-2/neu antibody was associated with the overexpression of HER-2/neu protein in tumors (99). Recent studies using HER2 autoantibodies to screen for breast cancer have yielded a median sensitivity and specificity of 17.4% and 94%, respectively (74). The analysis of HER2 expression alone is clearly not sufficient to screen for breast cancer. In terms of prognosis, studies have shown that high concentrations of anti-HER2 autoantibodies are associated with a good prognosis in patients with invasive breast cancer, possibly due to enhanced humoral immunity against breast cancer (100). Yukiko Tabuchi et al. also showed that the expression of HER2 autoantibodies was significantly associated with relapse-free survival of breast cancer (65).

### Other important autoantibodies

Heat-shock protein (HSP) plays an important role in breast cancer (101). In 1991, A. Thor et al. evaluated the amount of HSP-27 expression in human breast cancer and breast cancer cell lines and showed a relationship between HSP-27 overexpression and breast cancer invasiveness (102). However, subsequent studies have shown that HSP-27 and its autoantibodies are not suitable to be used as biomarkers for breast cancer screening due to low sensitivity (103). In 1993, A. Jameel et al. isolated and characterized a clone homologous to HSP-90 and found that its high expression was associated with early recurrence and reduced overall survival of breast cancer patients (104). S. E. Conroy et al. showed that HSP-90 autoantibodies are associated with metastasis of breast cancer (105). HSP-60 autoantibodies also contribute to the early diagnosis of breast cancer, especially ductal carcinoma *in situ* (DCIS) (106).

NY-ESO-1 autoantibodies have also been used in several studies for screening for breast cancer (38, 62). However, its sensitivity is very low, since only about 7% of breast cancer patients have positive serum NY-ESO-1 autoantibodies (34, 107). Autoantibodies of c-myc, BRCA1, BRCA2, cyclin B1, and survivin also have been used in several panels for screening for breast cancer (38, 45).

### Autoantibody panels

**Figure 2:**
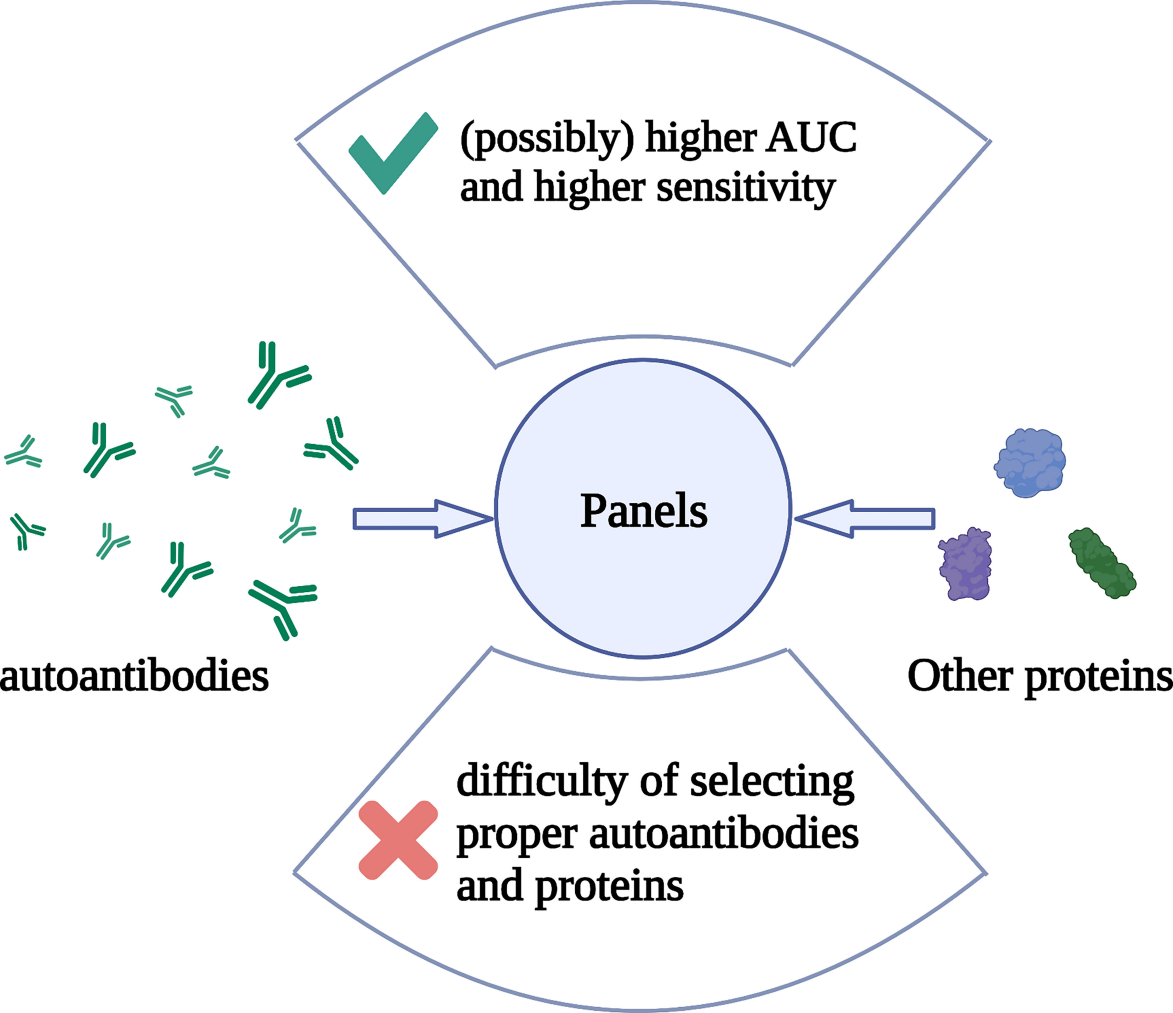
The composition, advantage, and problem of autoantibody panels. Multiple autoantibodies and/or other proteins constitutes a panel. Detection of these substances in peripheral blood can help in breast cancer screening. The advantage of panels is that they possibly help to obtain higher AUC and sensitivity than detecting for single autoantibodies, but the specificity may not be improved. The difficulty is that there is no recognized best method for selecting autoantibodies or other proteins that make up a panel. Created with BioRender.com.

Most autoantibodies are not capable of screening for breast cancer alone, but combining multiple autoantibodies or autoantibodies with other tumor biomarkers into a panel can improve the sensitivity and specificity. In recent years, Xuejun Dong et al. combined the classic biomarker CA 15-3 with FTH1 and hnRNPF autoantibodies into a panel and yielded a sensitivity of 89.3% and a specificity of 93.8% (108). A combination of multiple HSP and p53 autoantibodies yielded a sensitivity of 86% and a specificity of 100% (53). Although it is hard to do accurate mathematical statistics, we can see from **Table 1** that generally speaking, studies using panels have yielded a higher area under the ROC curve (AUC) and higher sensitivity, while the specificity does not seem to have been improved by panels (**Table 1** and **Figure 2**). However, the combination of more kinds of autoantibodies or proteins does not necessarily guarantee a higher specificity or sensitivity. A study detecting TOPO48 autoantibody alone has yielded a sensitivity of 100% and specificity of 76%, and the area under the AUC was 0.801, which is higher than that of many panels (59). To make good use of the advantages of panels, it is essential to select proper autoantibodies and proteins that make up the panels. However, there is no method recognized as the most effective for selecting the autoantibodies that comprise the panels.

## Methods used to detect autoantibodies

### Serological identification of antigens by recombinant expression cloning

**Figure 3:**
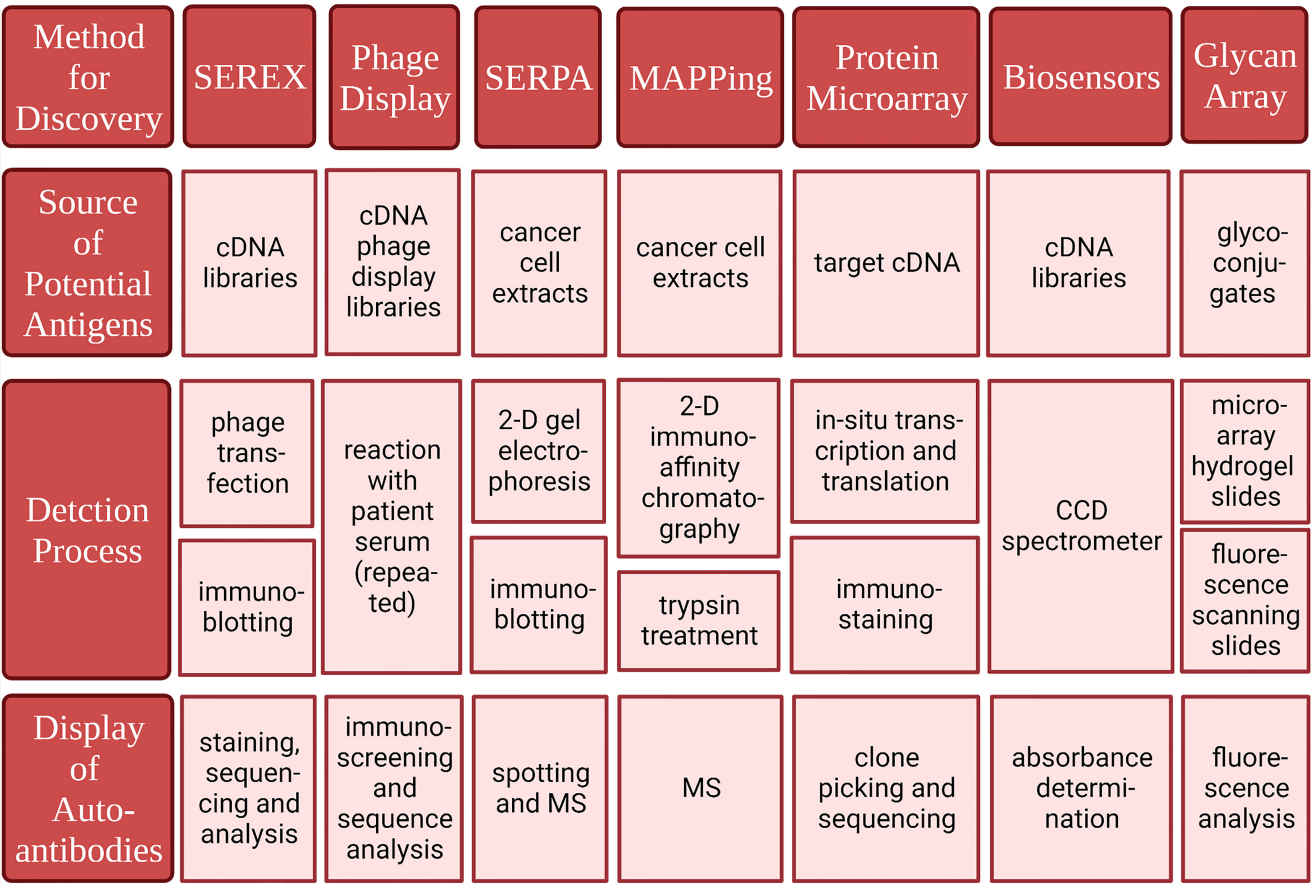
Methods for identifying tumor-associated antigens and correlated autoantibodies. Created with BioRender.com.

Michael Pfreundschuh et al. first used this technique in 1995 to extract mRNA and construct cDNA libraries from several kinds of cancer tissues (109). Diluted patient serum was reacted with recombinant proteins expressed in E. coli transfected with phage. Subsequently, serum antibodies binding to recombinant proteins were screened. Finally, staining was performed to show the results (109). This method does not require *in vitro* culture of cancer cell lines and CTL cells, thus avoiding MHC restriction, and can be used to detect autoantibodies against cancer cells from the serum of cancer patients (110). Moreover, SEREX can be used to detect a wide range of autoantibodies. SEREX has been used to detect peripheral blood autoantibodies in lung cancer, renal cell carcinoma, colon cancer, acute myeloid leukemia, hepatocellular carcinoma, gastric cancer, etc. (111–116). In breast cancer, ING1, LDH, fibulin-1, Topoisomerase-II-beta (TOPII), and Topoisomerase I binding protein (Topors) have been detected by SEREX (117–119). The disadvantage of SEREX is that the process is laborious and difficult to automate. Therefore, it can only be used for the discovery of new autoantibodies but not for the diagnosis of large numbers of patients and the screening of healthy people. Moreover, protein expression in phages does not involve posttranslational modification as it does in humans. Therefore, SEREX cannot be used to detect antibodies against autoantigens produced by posttranslational modifications. The characteristics and advantages of SEREX and the several methods introduced next are shown in (**Table 3** and **Figure 3**).

**Table 3:** Advantages and disadvantages of methods for detecting autoantibodies.

Method	Advantage(s)	Disadvantage(s)
SEREX	no need for *in vitro* cell culture, no MHC restriction; wide detection range	lack of automation; not suitable for mass screening; not suitable for posttranslational modification
Phage display	enrichment capacity, higher sensitivity and selectivity;smaller sample amount;screening of different phages in the same batch;	not suitable for posttranslational modification; not suitable for proteins that cannot be expressed on the surface of phages
SERPA	separating mixed proteins; suitable for posttranscriptional modifications and protein isotypes	not suitable for mass screening
MAPPing	suitable for low-abundance antigens	not suitable for mass screening
Protein Microarray	smaller sample amount; a direct platform for protein function analysis	difficulty of maintaining the tertiary structure of a protein
Biosensor (Nanobiosensors)	high selectivity, reproducibility, stability, sensitivity, and linearity	potential toxicities to the environment; miniaturization-induced unreliability; lack of automation; difficulty of integrating the nanostructured-based biosensors
Glycan Array	analyzing the interaction between biological macromolecules mediated by glycans quickly	not suitable for mass screening

### Phage display

Phage display uses cDNA derived from tumor tissue to construct cDNA phage display libraries. The phages are then reacted with diluted patient serum. The eluted phages are then amplified and screened again with the patient’s serum. This process is repeated several times (120, 121). This multiple screening processes have a strong enrichment capacity and thus leads to better sensitivity and selectivity than SEREX. Phage display also requires a smaller amount of patient serum and enables the screening of numerous different phages in the same batch, making detection more efficient (120, 121). Thus, phage display has many advantages over the use of a single screening method, such as SEREX. However, similar to SEREX, phage display cannot be used to detect antigens derived from posttranslational modifications. In addition, some proteins cannot be expressed on the surface of phages. Phage display has been used to detect autoantibodies in the breast, prostate, colon, stomach, hepatocellular carcinoma, etc. (119, 121–125). Xuejun Dong et al. combined CA 15-3 with heterogeneous nuclear ribonucleoproteins F (hnRNPF) and ferritin heavy chain (FTH1) detected through Phage display into a panel for breast cancer screening and achieved 89.3% sensitivity and 93.8% specificity (108). The proteins detected in the phage display can also be used to generate phage protein microarrays to establish autoantibody panels that can be used for screening (122, 124).

### Serological proteome analysis

Christoph S. Kladel et al. used SERPA in 2001 to identify serum autoantibodies in renal cell carcinoma (126). The SERPA process begins with the separation of mixed proteins in cancer tissue by two-dimensional gel electrophoresis and silver staining (126, 127). Then, immunoblotting was performed with the isolated protein and diluted serum from patients and healthy controls. Spots of binding are then revealed. Finally, the proteins are identified by mass spectrometry (MS) (126). SERPA has the advantage of being able to separate mixed proteins into single components by two-dimensional gel electrophoresis. This approach can be used to directly study tumor proteins extracted from tumor tissue and therefore can be used to detect a wide range of posttranscriptional modifications, protein isotypes, and so on. Additionally, unlike SEREX, SERPA does not require the construction of a cDNA library. SERPA has been used to detect serum autoantibodies in patients with gastric cancer, melanoma, gallbladder cancer, prostate cancer, thyroid cancer, lung cancer, etc. (128–133). Glucose-6-phosphate dehydrogenase (G6PD) was detected in breast cancer samples through SERPA (134). The detection range of SERPA is limited by its inability to be used to dissolve large, non-hydrophilic proteins, and it is difficult to detect low-abundance antigens with this method. As with SEREX, it is also difficult to assess a large number of samples with SERPA.

### Multiple affinity protein profiling

Julie Hardouin et al. detected serum autoantibodies in colon cancer using MAPPing (135). MAPPing involves two-dimensional immunoaffinity chromatography, trypsin treatment, and MS/MS analysis. In 2-D immunoaffinity chromatography, proteins that do not bind to antibodies in serum obtained from healthy controls are isolated, and then the antigens that could bind to IgG isolated from the patient’s serum are eluted and collected. Therefore, the possible tumor-associated antigens can be isolated (135). Two-dimensional immunoaffinity can be used to exclude a variety of high-abundance proteins that can react with antibodies in healthy control sera and enrich for low-abundance proteins, thus facilitating the detection of low-abundance tumor-associated antigens from confounding proteins (136).

### Protein microarray

The protein microarray can be used to present and assess hundreds of tumor antigens with low sample consumption. Anderson et al. applied a new protein chip technology, nucleic acid protein programmable array (NAPPA), with a three-phase sequential screening strategy. This approach involves printing the cDNA encoding the target gene on the substrate rather than the purified protein (137, 138). Within a few hours, the genes are transcribed and translated to produce the protein. This method minimizes protein degradation and preserves protein structure to the greatest extent possible, and the protein is prepared before the patient’s serum is tested. Finally, the specificity and sensitivity of 28 potential autoantibody biomarkers in the early detection of breast cancer were verified (139). Blixt et al. explored autoantibodies against abnormally glycosylated MUC1 and found that high levels of core3muc1 (glcnacb1-3galnac-muc1) and stnmuc1 (neuaca2,6galnac-muc1) glycotype autoantibody subsets were significantly related to a reduction in the incidence rate and an increase in metastasis time. The autoantibody response of patients with early breast cancer is highly correlated with age (37).

### Biosensors

Biosensors are sensitive to biological substances and convert them into electrical signals for detection. Because nanomaterials make biosensors more sensitive and more suitable for high-throughput analysis, there have been more studies on nanosensors in recent years. Masud et al. developed a gold-loaded nanoporous iron oxide nanocube (AU NPFE 2° 3 NC), which achieved good clinical adaptability in the detection of p53-specific autoantibodies (140). Feyzi Barnaji et al. generated an electrochemical biosensor with nanocomposites containing th/cs/ni(OH) 2 nps/ergo on the surface of a glassy carbon electrode and detected anti-p53 autoantibodies. The experimental results showed stability, reproducibility, and high sensitivity (141).

### Glycan array

A glycan array is a high-throughput device that can be used to detect autoantibodies against abnormal glycans (142, 143). Decades of research suggested that abnormal glycosylation was a sign of cancer (144). Abnormal glycan structure can cause an immune response earlier than disease symptoms arise and lead to the production of anti-glycan antibodies (145). Some groups have manufactured high-throughput devices to fix the sugar chain structure onto a glass surface to screen for anti-sugar chain antibodies in patient samples (37, 145, 146). Blixt et al. used a sugar chain array to identify anti-sugar chain antibodies against mucin 1 (MUC1) glycopeptide and found higher levels of MUC1 and cancer-related glycotypes in patients with early breast cancer (37).

### Validation methods

Single plex ELISAs are the most commonly used method to verify the presence of peripheral blood autoantibodies. Engvall, E. et al. were the first to use ELISA to measure IgG levels in rabbit serum (147). In 1985, Kostiala, A. A. et al. used ELISA to detect serum single-strand DNA (ssDNA) antibodies in patients with hematological malignancies who were followed up (148). In breast cancer, ELISA was first used to study serum p53 autoantibodies (76, 149). In addition to ELISA, Western blotting (WB) is also a commonly used assay.

## Conclusion and future directions

The analyses of existing autoantibodies lack sufficient specificity and sensitivity, most of which are not higher than mammography, and there is no standard for detecting autoantibodies for early cancer diagnosis. In addition, most studies have been about the relationship between autoantibodies and early cancer diagnosis. Only a few researchers have studied the relationship between autoantibodies and prognosis, and different studies of the same antibody sometimes have opposite results. Additionally, it is recommended that future autoantibody studies strictly follow the five-phase model and prospective sample collection retrospective evaluation (PRoBE) guidelines, which would enable autoantibody screening to be applied to clinical practice earlier (150, 151).

Although the analysis of peripheral blood autoantibodies is not sufficient when used alone to screen for breast cancer, this analysis can be used as a complement to mammography. The development and application of panels can improve the accuracy of screening for breast cancer with peripheral blood autoantibodies, and its effect is better than the effect of detecting a single autoantibody. Currently, most panels are limited to the combination of multiple autoantibodies. In the future, the combined analysis of autoantibodies and serum protein biomarkers or other components in peripheral blood can be studied, thus providing more possibilities for breast cancer screening (52). Some concerns remain about the analysis of peripheral blood autoantibodies. For example, the levels of some serum autoantibodies probably do not correlate well with the accumulation of corresponding antigens in cancer tissues, including p53 (77, 78). In addition, although many methods can detect peripheral blood autoantibodies, each method has limitations. More accurate and efficient detection methods are needed in the future. There are also studies examining the analysis of autoantibodies isolated from other body fluids, including saliva (152). Sample sources other than blood may be considered in the future.

In addition to being used for screening, peripheral blood autoantibodies can also contribute to the treatment and prognosis of breast cancer. Some autoantibodies have been linked to factors of prognosis, including survival rate, recurrence rate, and response to treatment. Testing for autoantibodies can help more accurately classify breast cancer, predict a patient’s risk, and determine how a patient is likely to respond to different treatment options so that the most effective option is selected. For example, the detection of serum anti-ER α autoantibodies is likely to help predict tamoxifen resistance in patients with ER-positive breast cancer, thus enabling appropriate treatment decisions (153). Recently, Rongrong Luo et al. identified five autoantibodies whose concentration differed in the serum of patients with different subtypes of breast cancer. The panel composed of the five autoantibodies can be used to discern triple-negative breast cancer from non-triple-negative breast cancer, and the AUC is 0.875 (63). At present, the specific mechanism of various autoantibodies and their corresponding antigens in the occurrence and development of breast cancer remains to be studied. Future research can focus on related proteins and their signaling pathways, thus providing new possibilities for the treatment of breast cancer.

## Author contributions

WY and QL designed the study. RY and YH drafted the manuscript. QL revised the manuscript. All authors contributed to the article and approved the submitted version.

## Funding

This study was funded by the Science and Technology Innovation Program of Hunan Province (Grant No. 2021SK2026), Health and Family Planning Commission of Hunan Province (Grant No. 20221J70143), and the National Natural Science Foundation of China (Grant No. 82270834 and 81873640).

## Conflict of interest

The authors declare that the research was conducted in the absence of any commercial or financial relationships that could be construed as a potential conflict of interest.

## Publisher’s note

All claims expressed in this article are solely those of the authors and do not necessarily represent those of their affiliated organizations, or those of the publisher, the editors and the reviewers. Any product that may be evaluated in this article, or claim that may be made by its manufacturer, is not guaranteed or endorsed by the publisher.

## References

[1] Lei S Zheng R Zhang S Wang S Chen R Sun K. Global patterns of breast cancer incidence and mortality: A population-based cancer registry data analysis from 2000 to 2020. Cancer Commun (London England). (2021) 41(11):1183–94. doi: 10.1002/cac2.12207 PMC8626596 34399040

[2] Siegel RL Miller KD Fuchs HE Jemal A. Cancer statistics, 2022. CA: Cancer J Clin (2022) 72(1):7–33. doi: 10.3322/caac.21708 35020204

[3] Miller KD Nogueira L Mariotto AB Rowland JH Yabroff KR Alfano CM. Cancer treatment and survivorship statistics, 2019. CA: Cancer J Clin (2019) 69(5):363–85. doi: 10.3322/caac.21565 31184787

[4] Bevers TB Helvie M Bonaccio E Calhoun KE Daly MB Farrar WB. Breast cancer screening and diagnosis, version 3.2018, NCCN clinical practice guidelines in oncology. J Natl Compr Cancer Network JNCCN (2018) 16(11):1362–89. doi: 10.6004/jnccn.2018.0083 30442736

[5] Oeffinger KC Fontham ET Etzioni R Herzig A Michaelson JS Shih YC. Breast cancer screening for women at average risk: 2015 guideline update from the American cancer society. JAMA (2015) 314(15):1599–614. doi: 10.1001/jama.2015.12783 PMC4831582 26501536

[6] Siu AL. Screening for breast cancer: U.S. preventive services task force recommendation statement. Ann Internal Med (2016) 164(4):279–96. doi: 10.7326/M15-2886 26757170

[7] Berry DA Cronin KA Plevritis SK Fryback DG Clarke L Zelen M. Effect of screening and adjuvant therapy on mortality from breast cancer. New Engl J Med (2005) 353(17):1784–92. doi: 10.1056/NEJMoa050518 16251534

[8] Hofvind S Ursin G Tretli S Sebuødegård S Møller B. Breast cancer mortality in participants of the Norwegian breast cancer screening program. Cancer. (2013) 119(17):3106–12. doi: 10.1002/cncr.28174 PMC4288930 23720226

[9] Coldman A Phillips N Wilson C Decker K Chiarelli AM Brisson J. Pan-Canadian study of mammography screening and mortality from breast cancer. J Natl Cancer Institute (2014) 106(11):dju261. doi: 10.1093/jnci/dju261 25274578

[10] Hofvind S Geller BM Skelly J Vacek PM. Sensitivity and specificity of mammographic screening as practised in Vermont and Norway. Br J Radiol (2012) 85(1020):e1226–32. doi: 10.1259/bjr/15168178 PMC3611728 22993383

[11] Wang J Gottschal P Ding L Veldhuizen DAV Lu W Houssami N. Mammographic sensitivity as a function of tumor size: A novel estimation based on population-based screening data. Breast (Edinburgh Scotland). (2021) 55:69–74. doi: 10.1016/j.breast.2020.12.003 33348148 PMC7753195

[12] Gøtzsche PC Jørgensen KJ. Screening for breast cancer with mammography. Cochrane Database systematic Rev (2013) 2013(6):Cd001877. doi: 10.1002/14651858.CD001877.pub5 PMC6464778 23737396

[13] Løberg M Lousdal ML Bretthauer M Kalager M. Benefits and harms of mammography screening. Breast Cancer Res BCR. (2015) 17(1):63. doi: 10.1186/s13058-015-0525-z 25928287 PMC4415291

[14] Elmore JG Barton MB Moceri VM Polk S Arena PJ Fletcher SW. Ten-year risk of false positive screening mammograms and clinical breast examinations. New Engl J Med (1998) 338(16):1089–96. doi: 10.1056/NEJM199804163381601 9545356

[15] Miller AB Wall C Baines CJ Sun P To T Narod SA. Twenty five year follow-up for breast cancer incidence and mortality of the Canadian national breast screening study: randomised screening trial. BMJ (2014) 348:g366. doi: 10.1136/bmj.g366 24519768 PMC3921437

[16] Nazari SS Mukherjee P. An overview of mammographic density and its association with breast cancer. Breast Cancer (Tokyo Japan). (2018) 25(3):259–67. doi: 10.1007/s12282-018-0857-5 PMC5906528 29651637

[17] Boyd NF Guo H Martin LJ Sun L Stone J Fishell E. Mammographic density and the risk and detection of breast cancer. New Engl J Med (2007) 356(3):227–36. doi: 10.1056/NEJMoa062790 17229950

[18] Wanders JO Holland K Veldhuis WB Mann RM Pijnappel RM Peeters PH. Volumetric breast density affects performance of digital screening mammography. Breast Cancer Res Treat (2017) 162(1):95–103. doi: 10.1007/s10549-016-4090-7 28012087 PMC5288416

[19] Axelrod D Smith J Kornreich D Grinstead E Singh B Cangiarella J. Breast cancer in young women. J Am Coll Surgeons. (2008) 206(6):1193–203. doi: 10.1016/j.jamcollsurg.2007.12.026 18501818

[20] Berg WA Zhang Z Lehrer D Jong RA Pisano ED Barr RG. Detection of breast cancer with addition of annual screening ultrasound or a single screening MRI to mammography in women with elevated breast cancer risk. JAMA (2012) 307(13):1394–404. doi: 10.1001/jama.2012.388 PMC3891886 22474203

[21] Cserni G Chmielik E Cserni B Tot T. The new TNM-based staging of breast cancer. Virchows Archiv an Int J pathology. (2018) 472(5):697–703. doi: 10.1007/s00428-018-2301-9 29380126

[22] Barrdahl M Canzian F Lindström S Shui I Black A Hoover RN. Association of breast cancer risk loci with breast cancer survival. Int J cancer. (2015) 137(12):2837–45. doi: 10.1002/ijc.29446 PMC4615576 25611573

[23] Houtsma D de Groot S Baak-Pablo R Kranenbarg EM Seynaeve CM van de Velde CJH. The variant T allele of PvuII in ESR1 gene is a prognostic marker in early breast cancer survival. Sci Rep (2021) 11(1):3249. doi: 10.1038/s41598-021-82002-z 33547330 PMC7864972

[24] Győrffy B Bottai G Fleischer T Munkácsy G Budczies J Paladini L. Aberrant DNA methylation impacts gene expression and prognosis in breast cancer subtypes. Int J cancer. (2016) 138(1):87–97. doi: 10.1002/ijc.29684 26174627

[25] Prat A Pineda E Adamo B Galván P Fernández A Gaba L. Clinical implications of the intrinsic molecular subtypes of breast cancer. Breast (Edinburgh Scotland). (2015) 24 Suppl 2:S26–35. doi: 10.1016/j.breast.2015.07.008 26253814

[26] Waks AG Winer EP. Breast cancer treatment: A review. JAMA (2019) 321(3):288–300. doi: 10.1001/jama.2018.19323 30667505

[27] Xiao Y Yu D. Tumor microenvironment as a therapeutic target in cancer. Pharmacol Ther (2021) 221:107753. doi: 10.1016/j.pharmthera.2020.107753 33259885 PMC8084948

[28] Suzuki H Graziano DF McKolanis J Finn OJ. T Cell-dependent antibody responses against aberrantly expressed cyclin B1 protein in patients with cancer and premalignant disease. Clin Cancer Res (2005) 11(4):1521–6. doi: 10.1158/1078-0432.CCR-04-0538 15746055

[29] Turnbull AR Turner DT Fraser JD Lloyd RS Lang CJ Wright R. Autoantibodies in early breast cancer: a stage-related phenomenon? Br J Cancer (1978) 38(3):461–3. doi: 10.1038/bjc.1978.230 PMC2009743 309335

[30] Lu H Ladd J Feng Z Wu M Goodell V Pitteri SJ. Evaluation of known oncoantibodies, HER2, p53, and cyclin B1, in prediagnostic breast cancer sera. Cancer Prev Res (Phila). (2012) 5(8):1036–43. doi: 10.1158/1940-6207.CAPR-11-0558 PMC3790582 22715141

[31] Henderson MC Silver M Tran Q Letsios EE Mulpuri R Reese DE. A noninvasive blood-based combinatorial proteomic biomarker assay to detect breast cancer in women over age 50 with BI-RADS 3, 4, or 5 assessment. Clin Cancer Res (2019) 25(1):142–9. doi: 10.1158/1078-0432.CCR-18-0843 30185421

[32] Reese DE Henderson MC Silver M Mulpuri R Letsios E Tran Q. Breast density does not impact the ability of videssa® breast to detect breast cancer in women under age 50. PLoS One (2017) 12(10):e0186198. doi: 10.1371/journal.pone.0186198 29069101 PMC5656317

[33] Jäger D Unkelbach M Frei C Bert F Scanlan MJ Jäger E. Identification of tumor-restricted antigens NY-BR-1, SCP-1, and a new cancer/testis-like antigen NW-BR-3 by serological screening of a testicular library with breast cancer serum. Cancer Immun (2002) 2:5. 12747750

[34] Stockert E Jäger E Chen YT Scanlan MJ Gout I Karbach J. A survey of the humoral immune response of cancer patients to a panel of human tumor antigens. J Exp Med (1998) 187(8):1349–54. doi: 10.1084/jem.187.8.1349 PMC2212223 9547346

[35] Joos TO Stoll D Templin MF. Miniaturised multiplexed immunoassays. Curr Opin Chem Biol (2002) 6(1):76–80. doi: 10.1016/S1367-5931(01)00289-7 11827827

[36] Wang J Figueroa JD Wallstrom G Barker K Park JG Demirkan G. Plasma autoantibodies associated with basal-like breast cancers. Cancer epidemiology Biomarkers Prev Publ Am Assoc Cancer Research cosponsored by Am Soc Prev Oncol (2015) 24(9):1332–40. doi: 10.1158/1055-9965.EPI-15-0047 PMC4560641 26070530

[37] Blixt O Bueti D Burford B Allen D Julien S Hollingsworth M. Autoantibodies to aberrantly glycosylated MUC1 in early stage breast cancer are associated with a better prognosis. Breast Cancer Res (2011) 13(2):R25. doi: 10.1186/bcr2841 21385452 PMC3219186

[38] Chapman C Murray A Chakrabarti J Thorpe A Woolston C Sahin U. Autoantibodies in breast cancer: their use as an aid to early diagnosis. Ann Oncol Off J Eur Soc Med Oncol (2007) 18(5):868–73. doi: 10.1093/annonc/mdm007 17347129

[39] Lacombe J Mangé A Jarlier M Bascoul-Mollevi C Rouanet P Lamy PJ. Identification and validation of new autoantibodies for the diagnosis of DCIS and node negative early-stage breast cancers. Int J Cancer. (2013) 132(5):1105–13. doi: 10.1002/ijc.27766 22886747

[40] Dong X Yang M Sun H Lü J Zheng Z Li Z. Combined measurement of CA 15-3 with novel autoantibodies improves diagnostic accuracy for breast cancer. Onco Targets Ther 4417. (2013) 6:273–9. doi: 10.2147/OTT.S43122 PMC3615893 23569391

[41] Heo CK Hwang HM Ruem A Yu DY Lee JY Yoo JS. Identification of a mimotope for circulating anti-cytokeratin 8/18 antibody and its usage for the diagnosis of breast cancer. Int J Oncol (2013) 42(1):65–74. doi: 10.3892/ijo.2012.1679 23128437 PMC3583721

[42] Lacombe J Mangé A Jarlier M Bascoul-Mollevi C Rouanet P Lamy PJ. 25721883. Int J cancer. (2013) 132(5):1105–13. doi: 10.1002/ijc.27766 22886747

[43] Ladd JJ Chao T Johnson MM Qiu J Chin A Israel R. Autoantibody signatures involving glycolysis and splicesome proteins precede a diagnosis of breast cancer among postmenopausal women. Cancer Res (2013) 73(5):1502–13. doi: 10.1158/0008-5472.CAN-12-2560 PMC4085738 23269276

[44] Yahalom G Weiss D Novikov I Bevers TB Radvanyi LG Liu M. An antibody-based blood test utilizing a panel of biomarkers as a new method for improved breast cancer diagnosis. Biomarkers cancer. (2013) 5:71–80. doi: 10.4137/BIC.S13236 PMC3855201 24324350

[45] Ye H Sun C Ren P Dai L Peng B Wang K. Mini-array of multiple tumor-associated antigens (TAAs) in the immunodiagnosis of breast cancer. Oncol letters. (2013) 5(2):663–8. doi: 10.3892/ol.2012.1062 PMC3573153 23420714

[46] Evans RL Pottala JV Egland KA. Classifying patients for breast cancer by detection of autoantibodies against a panel of conformation-carrying antigens. Cancer Prev Res (Philadelphia Pa). (2014) 7(5):545–55. doi: 10.1158/1940-6207.CAPR-13-0416 PMC4437507 24641868

[47] Lacombe J Mangé A Bougnoux AC Prassas I Solassol J. A multiparametric serum marker panel as a complementary test to mammography for the diagnosis of node-negative early-stage breast cancer and DCIS in young women. Cancer epidemiology Biomarkers Prev Publ Am Assoc Cancer Research cosponsored by Am Soc Prev Oncol (2014) 23(9):1834–42. doi: 10.1158/1055-9965.EPI-14-0267 24957886

[48] Liu W de la Torre IG Gutiérrez-Rivera MC Wang B Liu Y Dai L. Detection of autoantibodies to multiple tumor-associated antigens (TAAs) in the immunodiagnosis of breast cancer. Tumour Biol J Int Soc Oncodevelopmental Biol Med (2015) 36(2):1307–12. doi: 10.1007/s13277-014-2756-5 25355596

[49] Liu W Li Y Wang B Dai L Qian W Zhang JY. Autoimmune response to IGF2 mRNA-binding protein 2 (IMP2/p62) in breast cancer. Scandinavian J Immunol (2015) 81(6):502–7. doi: 10.1111/sji.12285 PMC4431935 25721883

[50] Schummer M Thorpe J Giraldez MD Bergan L Tewari M Urban N. Evaluating serum markers for hormone receptor-negative breast cancer. PLoS One (2015) 10(11):e0142911. doi: 10.1371/journal.pone.0142911 26565788 PMC4643893

[51] Gupta P Suman S Mishra M Mishra S Srivastava N Kumar V. Autoantibodies against TYMS and PDLIM1 proteins detected as circulatory signatures in Indian breast cancer patients. Proteomics Clin applications. (2016) 10(5):564–73. doi: 10.1002/prca.201500138 27068564

[52] Henderson MC Hollingsworth AB Gordon K Silver M Mulpuri R Letsios E. Integration of serum protein biomarker and tumor associated autoantibody expression data increases the ability of a blood-based proteomic assay to identify breast cancer. PLoS One (2016) 11(8):e0157692. doi: 10.1371/journal.pone.0157692 27508384 PMC4980010

[53] Shi L Gehin T Chevolot Y Souteyrand E Mangé A Solassol J. Anti-heat shock protein autoantibody profiling in breast cancer using customized protein microarray. Analytical bioanalytical Chem (2016) 408(5):1497–506. doi: 10.1007/s00216-015-9257-2 26715250

[54] Zuo X Chen L Liu L Zhang Z Zhang X Yu Q. Identification of a panel of complex autoantigens (LGALS3, PHB2, MUC1, and GK2) in combination with CA15-3 for the diagnosis of early-stage breast cancer. Tumour Biol J Int Soc Oncodevelopmental Biol Med (2016) 37(1):1309–17. doi: 10.1007/s13277-015-3932-y 26289852

[55] Kostianets O Shyyan M Antoniuk SV Filonenko V Kiyamova R. Panel of SEREX-defined antigens for breast cancer autoantibodies profile detection. Biomarkers Biochem Indic exposure response susceptibility to chemicals. (2017) 22(2):149–56. doi: 10.1080/1354750X.2016.1252952 27775439

[56] Liu Y Liao Y Xiang L Jiang K Li S Huangfu M. A panel of autoantibodies as potential early diagnostic serum biomarkers in patients with breast cancer. Int J Clin Oncol (2017) 22(2):291–6. doi: 10.1007/s10147-016-1047-0 27778118

[57] Chung JM Jung Y Kim YP Song J Kim S Kim JY. Identification of the thioredoxin-like 2 autoantibody as a specific biomarker for triple-negative breast cancer. J Breast Cancer. (2018) 21(1):87–90. doi: 10.4048/jbc.2018.21.1.87 29628988 PMC5880970

[58] Qiu C Wang P Wang B Shi J Wang X Li T. Establishment and validation of an immunodiagnostic model for prediction of breast cancer. Oncoimmunology. (2019) 9(1):1682382. doi: 10.1080/2162402X.2019.1682382 32002291 PMC6959442

[59] He X Jiang XH Yie KY Chen J Zhang JB Yie SM. An autoantibody against a 48-kd fragment of human DNA-topoiomerase I in breast cancer: Implication for diagnosis and prognosis, and antibody-dependent cellular cytotoxicity in vitro. Cell Immunol (2020) 347:104007. doi: 10.1016/j.cellimm.2019.104007 31732123

[60] Sumazaki M Ogata H Nabeya Y Kuwajima A Hiwasa T Shimada H. Multipanel assay of 17 tumor-associated antibodies for serological detection of stage 0/I breast cancer. Cancer Sci (2021) 112(5):1955–62. doi: 10.1111/cas.14860 PMC8088936 33605508

[61] Qiu C Wang B Wang P Wang X Ma Y Dai L. Identification of novel autoantibody signatures and evaluation of a panel of autoantibodies in breast cancer. Cancer Sci (2021) 112(8):3388–400. doi: 10.1111/cas.15021 PMC8353906 34115421

[62] Hong CQ Weng XF Huang XC Chu LY Wei LF Lin YW. A panel of tumor-associated autoantibodies for the detection of early-stage breast cancer. J Cancer. (2021) 12(9):2747–55. doi: 10.7150/jca.57019 PMC8040727 33854634

[63] Luo R Zheng C Song W Tan Q Shi Y Han X. High-throughput and multi-phases identification of autoantibodies in diagnosing early-stage breast cancer and subtypes. Cancer science. (2022) 113(2):770–83. doi: 10.1111/cas.15227 PMC8819333 34843149

[64] Özmen T Güllüoğlu BM Yegen C Soran A. Autoimmune thyroid disease and breast cancer prognosis. J Breast Health (2015) 11(2):67–71. doi: 10.5152/tjbh.2015.2462 28331694 PMC5351489

[65] Tabuchi Y Shimoda M Kagara N Naoi Y Tanei T Shimomura A. Protective effect of naturally occurring anti-HER2 autoantibodies on breast cancer. Breast Cancer Res Treat (2016) 157(1):55–63. doi: 10.1007/s10549-016-3801-4 27113738

[66] Demircan K Sun Q Bengtsson Y Seemann P Vallon-Christersson J Malmberg M. Autoimmunity to selenoprotein p predicts breast cancer recurrence. Redox Biol (2022) 53:102346. doi: 10.1016/j.redox.2022.102346 35636018 PMC9157254

[67] DeLeo AB Jay G Appella E Dubois GC Law LW Old LJ. Detection of a transformation-related antigen in chemically induced sarcomas and other transformed cells of the mouse. Proc Natl Acad Sci United States America. (1979) 76(5):2420–4. doi: 10.1073/pnas.76.5.2420 PMC383613 221923

[68] Jay G Khoury G DeLeo AB Dippold WG Old LJ. p53 transformation-related protein: detection of an associated phosphotransferase activity. Proc Natl Acad Sci United States America. (1981) 78(5):2932–6. doi: 10.1073/pnas.78.5.2932 PMC319473 6265926

[69] Crawford LV Pim DC Gurney EG Goodfellow P Taylor-Papadimitriou J. Detection of a common feature in several human tumor cell lines–a 53,000-dalton protein. Proc Natl Acad Sci United States America. (1981) 78(1):41–5. doi: 10.1073/pnas.78.1.41 PMC318985 6264441

[70] Baker SJ Fearon ER Nigro JM Hamilton SR Preisinger AC Jessup JM. Chromosome 17 deletions and p53 gene mutations in colorectal carcinomas. Sci (New York NY). (1989) 244(4901):217–21. doi: 10.1126/science.2649981 2649981

[71] Donehower LA Harvey M Slagle BL McArthur MJ Montgomery CA Jr. Butel JS. Mice deficient for p53 are developmentally normal but susceptible to spontaneous tumours. Nature. (1992) 356(6366):215–21. doi: 10.1038/356215a0 1552940

[72] Hernández Borrero LJ El-Deiry WS. Tumor suppressor p53: Biology, signaling pathways, and therapeutic targeting. Biochim Biophys Acta Rev cancer. (2021) 1876(1):188556. doi: 10.1016/j.bbcan.2021.188556 33932560 PMC8730328

[73] Crawford LV Pim DC Bulbrook RD. Detection of antibodies against the cellular protein p53 in sera from patients with breast cancer. Int J cancer. (1982) 30(4):403–8. doi: 10.1002/ijc.2910300404 6292117

[74] Xia J Shi J Wang P Song C Wang K Zhang J. Tumour-associated autoantibodies as diagnostic biomarkers for breast cancer: A systematic review and meta-analysis. Scandinavian J Immunol (2016) 83(6):393–408. doi: 10.1111/sji.12430 26991924

[75] Qiu C Wang P Wang B Shi J Wang X Li T. Establishment and validation of an immunodiagnostic model for prediction of breast cancer. Oncoimmunology. (2020) 9(1):1682382. doi: 10.1080/2162402X.2019.1682382 32002291 PMC6959442

[76] Mudenda B Green JA Green B Jenkins JR Robertson L Tarunina M. The relationship between serum p53 autoantibodies and characteristics of human breast cancer. Br J cancer. (1994) 69(6):1115–9. doi: 10.1038/bjc.1994.219 PMC1969453 8198980

[77] Balogh GA Mailo DA Corte MM Roncoroni P Nardi H Vincent E. Mutant p53 protein in serum could be used as a molecular marker in human breast cancer. Int J Oncol (2006) 28(4):995–1002. doi: 10.3892/ijo.28.4.995 16525651

[78] Vojtesek B Kovarik J Dolezalova H Nenutil R Havlis P Brentani RR. Absence of p53 autoantibodies in a significant proportion of breast cancer patients. Br J cancer. (1995) 71(6):1253–6. doi: 10.1038/bjc.1995.242 PMC2033849 7779720

[79] Lenner P Wiklund F Emdin SO Arnerlöv C Eklund C Hallmans G. Serum antibodies against p53 in relation to cancer risk and prognosis in breast cancer: a population-based epidemiological study. Br J Cancer (1999) 79(5-6):927–32. doi: 10.1038/sj.bjc.6690148 PMC2362685 10070892

[80] Sirotković-Skerlev M Plavetić ND Sedlić F Kuna SK Vrbanec D Belev B. Prognostic value of circulating bcl-2 and anti-p53 antibodies in patients with breast cancer: A long term follow-up (17.5 years). Cancer Biomarkers section A Dis Markers (2021) 30(1):95–104. doi: 10.3233/CBM-201497 PMC12499958 32986661

[81] Kulić A Sirotković-Skerlev M Jelisavac-Cosić S Herceg D Kovac Z Vrbanec D. Anti-p53 antibodies in serum: relationship to tumor biology and prognosis of breast cancer patients. Med Oncol (Northwood London England). (2010) 27(3):887–93. doi: 10.1007/s12032-009-9301-1 19763913

[82] Metcalfe S Wheeler TK Picken S Negus S Jo Milner A. P53 autoantibodies in 1006 patients followed up for breast cancer. Breast Cancer Res BCR. (2000) 2(6):438–43. doi: 10.1186/bcr91 PMC13921 11056691

[83] Gendler SJ Spicer AP. Epithelial mucin genes. Annu Rev Physiol (1995) 57:607–34. doi: 10.1146/annurev.ph.57.030195.003135 7778880

[84] Hattrup CL Gendler SJ. Structure and function of the cell surface (tethered) mucins. Annu Rev Physiol (2008) 70:431–57. doi: 10.1146/annurev.physiol.70.113006.100659 17850209

[85] Nath S Mukherjee P. MUC1: a multifaceted oncoprotein with a key role in cancer progression. Trends Mol Med (2014) 20(6):332–42. doi: 10.1016/j.molmed.2014.02.007 PMC5500204 24667139

[86] Gendler SJ. MUC1, the renaissance molecule. J Mammary Gland Biol Neoplasia. (2001) 6(3):339–53. doi: 10.1023/A:1011379725811 11547902

[87] Yolken RH Peterson JA Vonderfecht SL Fouts ET Midthun K Newburg DS. Human milk mucin inhibits rotavirus replication and prevents experimental gastroenteritis. J Clin Invest. (1992) 90(5):1984–91. doi: 10.1172/JCI116078 PMC443262 1331178

[88] Schroten H Hanisch FG Plogmann R Hacker J Uhlenbruck G Nobis-Bosch R. Inhibition of adhesion of s-fimbriated escherichia coli to buccal epithelial cells by human milk fat globule membrane components: a novel aspect of the protective function of mucins in the nonimmunoglobulin fraction. Infect Immun (1992) 60(7):2893–9. doi: 10.1128/iai.60.7.2893-2899.1992 PMC257251 1377184

[89] Rajabi H Jin C Ahmad R McClary C Joshi MD Kufe D. MUCIN 1 ONCOPROTEIN EXPRESSION IS SUPPRESSED BY THE miR-125b ONCOMIR. Genes Cancer. (2010) 1(1):62–8. doi: 10.1177/1947601909357933 PMC2923812 20729973

[90] Rakha EA Boyce RW Abd El-Rehim D Kurien T Green AR Paish EC. Expression of mucins (MUC1, MUC2, MUC3, MUC4, MUC5AC and MUC6) and their prognostic significance in human breast cancer. Mod Pathol (2005) 18(10):1295–304. doi: 10.1038/modpathol.3800445 15976813

[91] Whitehouse C Burchell J Gschmeissner S Brockhausen I Lloyd KO Taylor-Papadimitriou J. A transfected sialyltransferase that is elevated in breast cancer and localizes to the medial/trans-golgi apparatus inhibits the development of core-2-based O-glycans. J Cell Biol (1997) 137(6):1229–41. doi: 10.1083/jcb.137.6.1229 PMC2132526 9182658

[92] Picco G Julien S Brockhausen I Beatson R Antonopoulos A Haslam S. Over-expression of ST3Gal-I promotes mammary tumorigenesis. Glycobiology. (2010) 20(10):1241–50. doi: 10.1093/glycob/cwq085 PMC2934706 20534593

[93] Hermsen BB Verheijen RH Menko FH Gille JJ van Uffelen K Blankenstein MA. Humoral immune responses to MUC1 in women with a BRCA1 or BRCA2 mutation. Eur J Cancer. (2007) 43(10):1556–63. doi: 10.1016/j.ejca.2007.04.007 17532207

[94] Burford B Gentry-Maharaj A Graham R Allen D Pedersen JW Nudelman AS. Autoantibodies to MUC1 glycopeptides cannot be used as a screening assay for early detection of breast, ovarian, lung or pancreatic cancer. Br J cancer. (2013) 108(10):2045–55. doi: 10.1038/bjc.2013.214 PMC3670483 23652307

[95] Schechter AL Stern DF Vaidyanathan L Decker SJ Drebin JA Greene MI. The neu oncogene: an erb-b-related gene encoding a 185,000-Mr tumour antigen. Nature. (1984) 312(5994):513–6. doi: 10.1038/312513a0 6095109

[96] Schechter AL Hung MC Vaidyanathan L Weinberg RA Yang-Feng TL Francke U. The neu gene: an erbB-homologous gene distinct from and unlinked to the gene encoding the EGF receptor. Sci (New York NY). (1985) 229(4717):976–8. doi: 10.1126/science.2992090 2992090

[97] Coussens L Yang-Feng TL Liao YC Chen E Gray A McGrath J. Tyrosine kinase receptor with extensive homology to EGF receptor shares chromosomal location with neu oncogene. Sci (New York NY). (1985) 230(4730):1132–9. doi: 10.1126/science.2999974 2999974

[98] Natali PG Nicotra MR Bigotti A Venturo I Slamon DJ Fendly BM. Expression of the p185 encoded by HER2 oncogene in normal and transformed human tissues. Int J cancer. (1990) 45(3):457–61. doi: 10.1002/ijc.2910450314 1968437

[99] Disis ML Pupa SM Gralow JR Dittadi R Menard S Cheever MA. High-titer HER-2/neu protein-specific antibody can be detected in patients with early-stage breast cancer. J Clin Oncol Off J Am Soc Clin Oncol (1997) 15(11):3363–7. doi: 10.1200/JCO.1997.15.11.3363 9363867

[100] Sato Y Shimoda M Sota Y Miyake T Tanei T Kagara N. Enhanced humoral immunity in breast cancer patients with high serum concentration of anti-HER2 autoantibody. Cancer Med (2021) 10(4):1418–30. doi: 10.1002/cam4.3742 PMC7926031 33506656

[101] Alberti G Vergilio G Paladino L Barone R Cappello F Conway de Macario E. The chaperone system in breast cancer: Roles and therapeutic prospects of the molecular chaperones Hsp27, Hsp60, Hsp70, and Hsp90. Int J Mol Sci (2022) 23(14):7792. doi: 10.3390/ijms23147792 35887137 PMC9324353

[102] Thor A Benz C Moore D 2nd Goldman E Edgerton S Landry J. Stress response protein (srp-27) determination in primary human breast carcinomas: clinical, histologic, and prognostic correlations. J Natl Cancer Institute (1991) 83(3):170–8. doi: 10.1093/jnci/83.3.170 1988702

[103] Fanelli MA Cuello Carrión FD Dekker J Schoemaker J Ciocca DR. Serological detection of heat shock protein hsp27 in normal and breast cancer patients. Cancer epidemiology Biomarkers Prev Publ Am Assoc Cancer Research cosponsored by Am Soc Prev Oncol (1998) 7(9):791–5. Available at: https://aacrjournals.org/cebp/article/7/9/791/108718/Serological-detection-of-heat-shock-protein-hsp27. 9752987

[104] Jameel A Law M Coombes R Luqmani Y. Significance of heat-shock protein-90 as a prognostic indicator in breast-cancer. Int J Oncol (1993) 2(6):1075–80. doi: 10.3892/ijo.2.6.1075 21573675

[105] Conroy SE Gibson SL Brunström G Isenberg D Luqmani Y Latchman DS. Autoantibodies to 90 kD heat-shock protein in sera of breast cancer patients. Lancet (London England) (1995) 345(8942):126. doi: 10.1016/S0140-6736(95)90090-X 7815863

[106] Desmetz C Bibeau F Boissière F Bellet V Rouanet P Maudelonde T. Proteomics-based identification of HSP60 as a tumor-associated antigen in early stage breast cancer and ductal carcinoma in situ. J Proteome Res (2008) 7(9):3830–7. doi: 10.1021/pr800130d 18683965

[107] Oshima Y Shimada H Yajima S Nanami T Matsushita K Nomura F. NY-ESO-1 autoantibody as a tumor-specific biomarker for esophageal cancer: screening in 1969 patients with various cancers. J gastroenterology. (2016) 51(1):30–4. doi: 10.1007/s00535-015-1078-8 25906289

[108] Dong X Yang M Sun H Lü J Zheng Z Li Z. Combined measurement of CA 15-3 with novel autoantibodies improves diagnostic accuracy for breast cancer. OncoTargets Ther (2013) 6:273–9. doi: 10.2147/OTT.S43122 PMC3615893 23569391

[109] Sahin U Türeci O Schmitt H Cochlovius B Johannes T Schmits R. Human neoplasms elicit multiple specific immune responses in the autologous host. Proc Natl Acad Sci United States America. (1995) 92(25):11810–3. doi: 10.1073/pnas.92.25.11810 PMC40492 8524854

[110] Obata Y TA T Tamaki H Tominaga S Murai H Iwase T. Identification of cancer antigens in breast cancer by the SEREX expression cloning method. Breast Cancer (Tokyo Japan). (1999) 6(4):305–11. doi: 10.1007/BF02966445 11091735

[111] Güre AO Stockert E Scanlan MJ Keresztes RS Jäger D Altorki NK. Serological identification of embryonic neural proteins as highly immunogenic tumor antigens in small cell lung cancer. Proc Natl Acad Sci United States America. (2000) 97(8):4198–203. doi: 10.1073/pnas.97.8.4198 PMC18195 10760287

[112] Scanlan MJ Gordan JD Williamson B Stockert E Bander NH Jongeneel V. Antigens recognized by autologous antibody in patients with renal-cell carcinoma. Int J cancer. (1999) 83(4):456–64. doi: 10.1002/(SICI)1097-0215(19991112)83:4<456::AID-IJC4>3.0.CO;2-5 10508479

[113] Scanlan MJ Chen YT Williamson B Gure AO Stockert E Gordan JD. Characterization of human colon cancer antigens recognized by autologous antibodies. Int J cancer. (1998) 76(5):652–8. doi: 10.1002/(SICI)1097-0215(19980529)76:5<652::AID-IJC7>3.0.CO;2-P 9610721

[114] Greiner J Ringhoffer M Simikopinko O Szmaragowska A Huebsch S Maurer U. Simultaneous expression of different immunogenic antigens in acute myeloid leukemia. Exp hematology. (2000) 28(12):1413–22. doi: 10.1016/S0301-472X(00)00550-6 11146163

[115] Stenner-Liewen F Luo G Sahin U Tureci O Koslovski M Kautz I. Definition of tumor-associated antigens in hepatocellular carcinoma. Cancer epidemiology Biomarkers Prev Publ Am Assoc Cancer Research cosponsored by Am Soc Prev Oncol (2000) 9(3):285–90. Available at: https://aacrjournals.org/cebp/article/9/3/285/283071/Definition-of-Tumor-associated-Antigens-in. 10750667

[116] Obata Y Takahashi T Sakamoto J Tamaki H Tominaga S Hamajima N. SEREX analysis of gastric cancer antigens. Cancer chemotherapy Pharmacol (2000) 46 Suppl:S37–42. doi: 10.1007/PL00014048 10950146

[117] Jäger D Stockert E Scanlan MJ Güre AO Jäger E Knuth A. Cancer-testis antigens and ING1 tumor suppressor gene product are breast cancer antigens: characterization of tissue-specific ING1 transcripts and a homologue gene. Cancer Res (1999) 59(24):6197–204. Available at: https://aacrjournals.org/cancerres/article/59/24/6197/505752/Cancer-Testis-Antigens-and-ING1-Tumor-Suppressor. 10626813

[118] Forti S Scanlan MJ Invernizzi A Castiglioni F Pupa S Agresti R. Identification of breast cancer-restricted antigens by antibody screening of SKBR3 cDNA library using a preselected patient's serum. Breast Cancer Res Treat (2002) 73(3):245–56. doi: 10.1023/A:1015854415746 12160330

[119] Minenkova O Pucci A Pavoni E De Tomassi A Fortugno P Gargano N. Identification of tumor-associated antigens by screening phage-displayed human cDNA libraries with sera from tumor patients. Int J cancer. (2003) 106(4):534–44. doi: 10.1002/ijc.11269 12845649

[120] Sioud M Hansen M Dybwad A. Profiling the immune responses in patient sera with peptide and cDNA display libraries. Int J Mol Med (2000) 6(2):123–8. doi: 10.3892/ijmm.6.2.123 10891554

[121] Sioud M Hansen MH. Profiling the immune response in patients with breast cancer by phage-displayed cDNA libraries. Eur J Immunol (2001) 31(3):716–25. doi: 10.1002/1521-4141(200103)31:3<716::AID-IMMU716>3.0.CO;2-9 11241275

[122] Wang X Yu J Sreekumar A Varambally S Shen R Giacherio D. Autoantibody signatures in prostate cancer. New Engl J Med (2005) 353(12):1224–35. doi: 10.1056/NEJMoa051931 16177248

[123] Ran Y Hu H Zhou Z Yu L Sun L Pan J. Profiling tumor-associated autoantibodies for the detection of colon cancer. Clin Cancer Res an Off J Am Assoc Cancer Res (2008) 14(9):2696–700. doi: 10.1158/1078-0432.CCR-07-2021 18451234

[124] Zayakin P Ancāns G Siliņa K Meistere I Kalniņa Z Andrejeva D. Tumor-associated autoantibody signature for the early detection of gastric cancer. Int J cancer. (2013) 132(1):137–47. doi: 10.1002/ijc.27667 22684876

[125] Liu H Zhang J Wang S Pang Z Wang Z Zhou W. Screening of autoantibodies as potential biomarkers for hepatocellular carcinoma by using T7 phase display system. Cancer Epidemiol (2012) 36(1):82–8. doi: 10.1016/j.canep.2011.04.001 22018955

[126] Klade CS Voss T Krystek E Ahorn H Zatloukal K Pummer K. Identification of tumor antigens in renal cell carcinoma by serological proteome analysis. Proteomics. (2001) 1(7):890–8. doi: 10.1002/1615-9861(200107)1:7<890::AID-PROT890>3.0.CO;2-Z 11503213

[127] Lee PY Saraygord-Afshari N Low TY. The evolution of two-dimensional gel electrophoresis - from proteomics to emerging alternative applications. J Chromatogr A. (2020) 1615:460763. doi: 10.1016/j.chroma.2019.460763 31836310

[128] Qin J Yang Q Ye H Wang K Zhang M Zhu J. Using serological proteome analysis to identify and evaluate anti-GRP78 autoantibody as biomarker in the detection of gastric cancer. J Oncol (2020) 2020:9430737. doi: 10.1155/2020/9430737 33381181 PMC7762641

[129] Suzuki A Iizuka A Komiyama M Takikawa M Kume A Tai S. Identification of melanoma antigens using a serological proteome approach (SERPA). Cancer Genomics proteomics. (2010) 7(1):17–23. Available at: https://cgp.iiarjournals.org/content/7/1/17.long. 20181627

[130] Akhtar J Priya R Jain V Sakhuja P Agarwal AK Goyal S. Immunoproteomics approach revealed elevated autoantibody levels against ANXA1 in early stage gallbladder carcinoma. BMC cancer. (2020) 20(1):1175. doi: 10.1186/s12885-020-07676-6 33261560 PMC7709428

[131] Dai L Li J Xing M Sanchez TW Casiano CA Zhang JY. Using serological proteome analysis to identify serum anti-nucleophosmin 1 autoantibody as a potential biomarker in European-American and African-American patients with prostate cancer. Prostate. (2016) 76(15):1375–86. doi: 10.1002/pros.23217 27418398

[132] Belousov PV Afanasyeva MA Gubernatorova EO Bogolyubova AV Uvarova AN Putlyaeva LV. Multi-dimensional immunoproteomics coupled with *in vitro* recapitulation of oncogenic NRAS(Q61R) identifies diagnostically relevant autoantibody biomarkers in thyroid neoplasia. Cancer letters. (2019) 467:96–106. doi: 10.1016/j.canlet.2019.07.013 31326556

[133] Dai L Li J Tsay JJ Yie TA Munger JS Pass H. Identification of autoantibodies to ECH1 and HNRNPA2B1 as potential biomarkers in the early detection of lung cancer. Oncoimmunology. (2017) 6(5):e1310359. doi: 10.1080/2162402X.2017.1310359 28638733 PMC5467997

[134] Canelle L Bousquet J Pionneau C Deneux L Imam-Sghiouar N Caron M. An efficient proteomics-based approach for the screening of autoantibodies. J Immunol Methods (2005) 299(1-2):77–89. doi: 10.1016/j.jim.2005.01.015 15914192

[135] Hardouin J Lasserre JP Canelle L Duchateau M Vlieghe C Choquet-Kastylevsky G. Usefulness of autoantigens depletion to detect autoantibody signatures by multiple affinity protein profiling. J separation science. (2007) 30(3):352–8. doi: 10.1002/jssc.200600324 17396593

[136] Wu C Duan J Liu T Smith RD Qian WJ. Contributions of immunoaffinity chromatography to deep proteome profiling of human biofluids. J Chromatogr B Analytical Technol Biomed Life Sci (2016) 1021:57–68. doi: 10.1016/j.jchromb.2016.01.015 PMC4862894 26868616

[137] Ramachandran N Anderson KS Raphael JV Hainsworth E Sibani S Montor WR. Tracking humoral responses using self assembling protein microarrays. Proteomics Clin Appl (2008) 2(10-11):1518–27. doi: 10.1002/prca.200800034 PMC3158030 21136799

[138] Ramachandran N Hainsworth E Bhullar B Eisenstein S Rosen B Lau AY. Self-assembling protein microarrays. Science. (2004) 305(5680):86–90. doi: 10.1126/science.1097639 15232106

[139] Anderson KS Sibani S Wallstrom G Qiu J Mendoza EA Raphael J. Protein microarray signature of autoantibody biomarkers for the early detection of breast cancer. J Proteome Res (2011) 10(1):85–96. doi: 10.1021/pr100686b 20977275 PMC3158028

[140] Masud MK Yadav S Islam MN Nguyen NT Salomon C Kline R. Gold-loaded nanoporous ferric oxide nanocubes with peroxidase-mimicking activity for electrocatalytic and colorimetric detection of autoantibody. Anal Chem (2017) 89(20):11005–13. doi: 10.1021/acs.analchem.7b02880 28892622

[141] Feyzi-Barnaji B Dinarvand R Salehzadeh H Arkan E Salimi A Nili F. Construction of a ternary nano-architecture based graphene oxide sheets, toward electrocatalytic determination of tumor-associated anti-p53 autoantibodies in human serum. Talanta. (2021) 230:122276. doi: 10.1016/j.talanta.2021.122276 33934760

[142] Liang PH Wu CY Greenberg WA Wong CH. Glycan arrays: biological and medical applications. Curr Opin Chem Biol (2008) 12(1):86–92. doi: 10.1016/j.cbpa.2008.01.031 18258211 PMC7108407

[143] Liang CH Wu CY. Glycan array: a powerful tool for glycomics studies. Expert Rev Proteomics. (2009) 6(6):631–45. doi: 10.1586/epr.09.82 19929609

[144] Munkley J Elliott DJ. Hallmarks of glycosylation in cancer. Oncotarget. (2016) 7(23):35478–89. doi: 10.18632/oncotarget.8155 PMC5085245 27007155

[145] Wandall HH Blixt O Tarp MA Pedersen JW Bennett EP Mandel U. Cancer biomarkers defined by autoantibody signatures to aberrant O-glycopeptide epitopes. Cancer Res (2010) 70(4):1306–13. doi: 10.1158/0008-5472.CAN-09-2893 PMC5538776 20124478

[146] Wang CC Huang YL Ren CT Lin CW Hung JT Yu JC. Glycan microarray of globo h and related structures for quantitative analysis of breast cancer. Proc Natl Acad Sci U S A. (2008) 105(33):11661–6. doi: 10.1073/pnas.0804923105 PMC2575271 18689688

[147] Engvall E Perlmann P. Enzyme-linked immunosorbent assay (ELISA). Quantitative assay immunoglobulin G. Immunochemistry. (1971) 8(9):871–4. doi: 10.1016/0019-2791(71)90454-x 5135623

[148] Kostiala AA Gripenberg M Elonen E Gripenberg G Kostiala I. Follow-up of antibodies against single-stranded DNA in patients with haematological malignancies. Clin Exp Immunol (1985) 61(1):15–23. 3876179 PMC1577234

[149] Green JA Mudenda B Jenkins J Leinster SJ Tarunina M Green B. Serum p53 auto-antibodies: incidence in familial breast cancer. Eur J Cancer (1994) 30a(5):580–4. doi: 10.1016/0959-8049(94)90523-1 8080669

[150] Pepe MS Feng Z Janes H Bossuyt PM Potter JD. Pivotal evaluation of the accuracy of a biomarker used for classification or prediction: standards for study design. J Natl Cancer Inst (2008) 100(20):1432–8. doi: 10.1093/jnci/djn326 PMC2567415 18840817

[151] Pepe MS Etzioni R Feng Z Potter JD Thompson ML Thornquist M. Phases of biomarker development for early detection of cancer. J Natl Cancer Inst (2001) 93(14):1054–61. doi: 10.1093/jnci/93.14.1054 11459866

[152] Arif S Qudsia S Urooj S Chaudry N Arshad A Andleeb S. Blueprint of quartz crystal microbalance biosensor for early detection of breast cancer through salivary autoantibodies against ATP6AP1. Biosensors bioelectronics. (2015) 65:62–70. doi: 10.1016/j.bios.2014.09.088 25461139

[153] Maselli A Parlato S Puglisi R Raggi C Spada M Macchia D. Autoantibodies specific to ERα are involved in tamoxifen resistance in hormone receptor positive breast cancer. Cells (2019) 8(7):750. doi: 10.3390/cells8070750 31331091 PMC6678306

